# Enhancing the sialylation of recombinant EPO produced in CHO cells via the inhibition of glycosphingolipid biosynthesis

**DOI:** 10.1038/s41598-017-13609-4

**Published:** 2017-10-12

**Authors:** Chan-Yeong Kwak, Seung-Yeol Park, Chung-Geun Lee, Nozomu Okino, Makoto Ito, Jung Hoe Kim

**Affiliations:** 1 Department of Biological Sciences, Korea Advanced Institute of Science and Technology, 335 Gwahangno, Yuseong-gu, Daejeon, 305-701 Republic of Korea; 2 Division of Rheumatology, Immunology and Allergy, Brigham and Women’s Hospital, and Department of Medicine, Harvard Medical School, Boston, MA 02115 USA; 3 Department of Bioscience and Biotechnology, Graduate School of Bioresource and Bioenvironmental Sciences, Kyushu University, 6-10-1, Hakozaki, Higashi-ku, Fukuoka, 812-8581 Japan

## Abstract

Sialylation regulates the *in vivo* half-life of recombinant therapeutic glycoproteins, affecting their therapeutic efficacy. Levels of the precursor molecule cytidine monophospho-N-acetylneuraminic acid (CMP-Neu5Ac) are considered a limiting factor in the sialylation of glycoproteins. Here, we show that by reducing the amount of intracellular CMP-Neu5Ac consumed for glycosphingolipid (GSL) biosynthesis, we can increase the sialylation of recombinant human erythropoietin (rhEPO) produced in CHO cells. Initially, we found that treating CHO cells with a potent inhibitor of GSL biosynthesis increases the sialylation of the rhEPO they produce. Then, we established a stable CHO cell line that produces rhEPO in the context of repression of the key GSL biosynthetic enzyme UDP-glucose ceramide glucosyltransferase (*UGCG*). These *UGCG*-depleted cells show reduced levels of gangliosides and significantly elevated levels of rhEPO sialylation. Upon further analysis of the resulting N-glycosylation pattern, we discovered that the enhanced rhEPO sialylation could be attributed to a decrease in neutral and mono-sialylated N-glycans and an increase in di-sialylated N-glycans. Our results suggest that the therapeutic efficacy of rhEPO produced in CHO cells can be improved by shunting intracellular CMP-Neu5Ac away from GSL biosynthesis and toward glycoprotein sialylation.

## Introduction

Many of the therapeutic bio-drugs currently in commercial production are sialylated glycoproteins^1^. Sialic acid is a terminal sugar residue on the N- and O-linked glycosylation chains attached to many glycoproteins. Since secreted therapeutic glycoproteins are degraded by hepatocytes after being recognized by the asialoglycoprotein receptor (ASGPR), enhanced sialylation of these therapeutic glycoproteins can increase their *in vivo* circulating half-life^2,3^. Thus, because it will likely enhance therapeutic efficacy, there is considerable interest in developing methods for enhancing the sialylation of recombinant glycoproteins.

Early attempts at promoting sialylation focused on the over-expression of sialylation-related enzymes^4,5^, but these efforts quickly hit a ceiling^6^. It became clear that the intracellular level of CMP-Neu5Ac, a precursor required for sialic acid biosynthesis, was limiting^7–9^. Attempts to increase intracellular CMP-Neu5Ac have included supplementation of the CMP-Neu5Ac biosynthetic precursor ManNAc^10^, over-expression of the CMP-Neu5Ac transporter (CST)^11,12^, and over-expression of mutant UDP-N-acetyl-glucosamine-2-epimerase (GNE) to bypass CMP-Neu5Ac-mediated feedback inhibition^13,14^. Recently, we also found a further increase in rhEPO sialylation upon over-expression of α2,3-sialyltransferase (ST) in CHO cells expressing mutant GNE and CST^15^.

In addition to its role in glycoprotein biology, sialic acid is also attached to glycosphingolipids (GSLs) including those of the Ganglio-series (GlcNAcβ4Galβ4GlcβCer), Globo-series (Galα4Galβ4GlcβCer), Lacto-series (GlcNAcβ3Galβ4GlcβCer), and Neolacto-series (Galβ4GlcNAcβ3Galβ4GlcβCer)^16^. Sialylation occurs when CMP-Neu5Ac is translocated to the Golgi complex. There, ST catalyzes the transfer of Neu5Ac to a terminal galactosyl residue^17^. The inhibition of CMP-Neu5Ac translocation to the Golgi reduces the sialylation of both glycoproteins and GSLs, suggesting CMP-Neu5Ac is required for both^18^. It is unclear, however, whether GSL sialylation would correlate with glycoprotein sialylation. This is the question we attempted to address in the present study. Indeed, we found that treating CHO cells with a potent inhibitor of GSL biosynthesis increases their sialylation of rhEPO. Subsequently, we established a stable cell line with reduced expression of the *UGCG* gene. These cells too show enhanced rhEPO sialylation. After performing a more detailed N-glycan analysis, we found that *UGCG*-depleted CHO cells produce rhEPO with increased levels of di-sialylated N-glycan.

## Results

### EtDO-P4 treatment enhances sialylation

**Figure 1:**
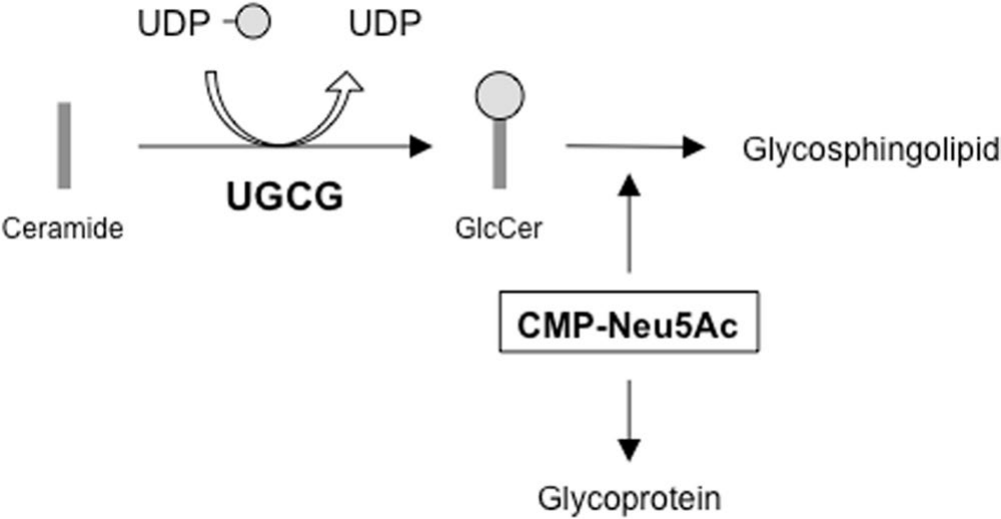
Ganglioside biosynthesis schematic. *UGCG* catalyzes the first step of GSL biosynthesis. CMP-Neu5Ac is a sialylation precursor in both glycoproteins and GSLs.

**Figure 2:**
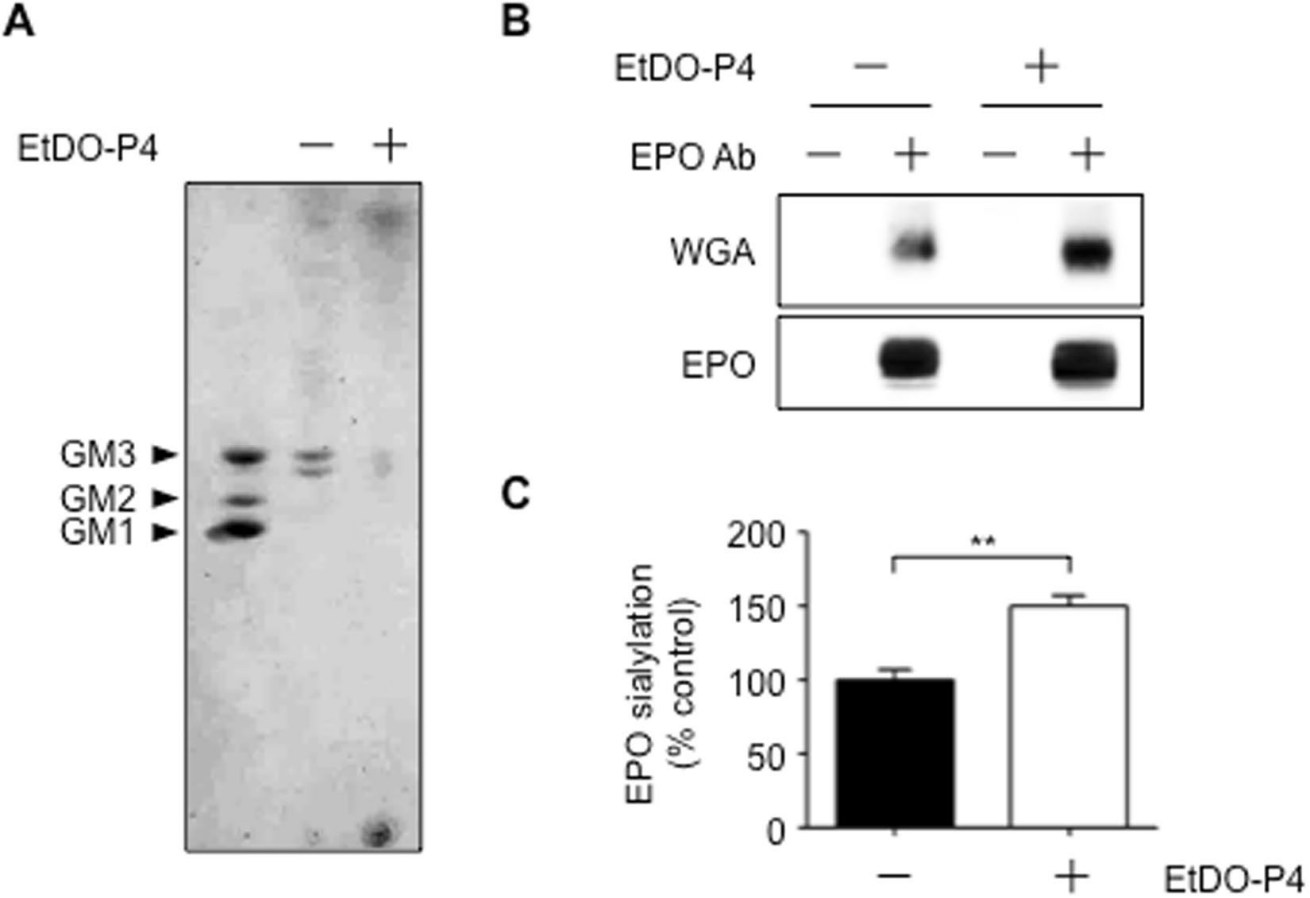
EtDO-P4 treatment increases rhEPO sialylation. (**A**) Total GSLs extracted from cells were subjected to TLC followed by staining with orcinol/sulfuric acid. Lane 1, GSL standards; Lane 2, GSLs extracted from EC2-1H9 cells; Lane 3, GSLs extracted from EC2-1H9 treated with EtDO-P4. (**B**) rhEPO was purified using immunoprecipitation with an anti-EPO antibody. Sialylation was determined using WGA lectin. (**C**) Quantification of data in B; n = 3. **P < 0.01 (Two-tailed Student’s *t*-test). Data are presented as means ± S.E.M.

To investigate the effect of GSL biosynthesis on glycoprotein sialylation, we used a chemical inhibitor of GSL biosynthesis. Ceramide glucosyltransferase (CGT) catalyzes the transfer of the glucose moiety from UDP-glucose to ceramide to produce glucosylceramide (GlcCer). GlcCer, in turn, acts as the GSL core (Fig. 1). EtDO-P4 (D-*threo*-ethylenedioxyphenyl-2-palmitoylamino-3-pyrrolidino-propanol) inhibits CGT and is, therefore, capable of reducing GSL biosynthesis in various cells^19,20^. After treating rhEPO-producing EC2-1H9 CHO cells with EtDO-P4, we measured their GSL biosynthesis using thin-layer chromatography (TLC) as previously described^21^. We found that ganglioside GM3 is the predominant GSL in CHO cells, with gangliosides GM1 and GM2 making up only minor fractions (Fig. 2A). After EtDO-P4 treatment, however, we observed a significant reduction in GSL biosynthesis (Fig. 2A). Although ganglioside GM3 reportedly induces cell proliferation in various cancer cells by activating cell surface receptors like EGFR and integrins^22^, EtDO-P4 does not alter cell proliferation in EC2-1H9 CHO cells (Fig. S1).

We previously reported that over-expression of glycosylation enzymes in CHO cells producing rhEPO leads to increased rhEPO sialylation^6,23^. To determine whether the inhibition of GSL biosynthesis can enhance the sialylation of therapeutic glycoproteins, we cultured EC2-1H9 CHO cells producing rhEPO as previously described^23^. We then purified rhEPO they produced via immunoprecipitation with an anti-EPO antibody and measured its sialylation level using lectin blotting with wheat germ agglutinin (WGA). This technique is specific for terminal sialic acids^24^. Indeed, we found that although EtDO-P4 treatment slightly reduces rhEPO production, it increases rhEPO sialylation by 50% (Fig. 2B and C). This suggests reducing the amount of CMP-Neu5Ac consumed by GSL production increases rhEPO sialylation.

### Transient inhibition of GSL biosynthesis enhances rhEPO sialylation

**Figure 3:**
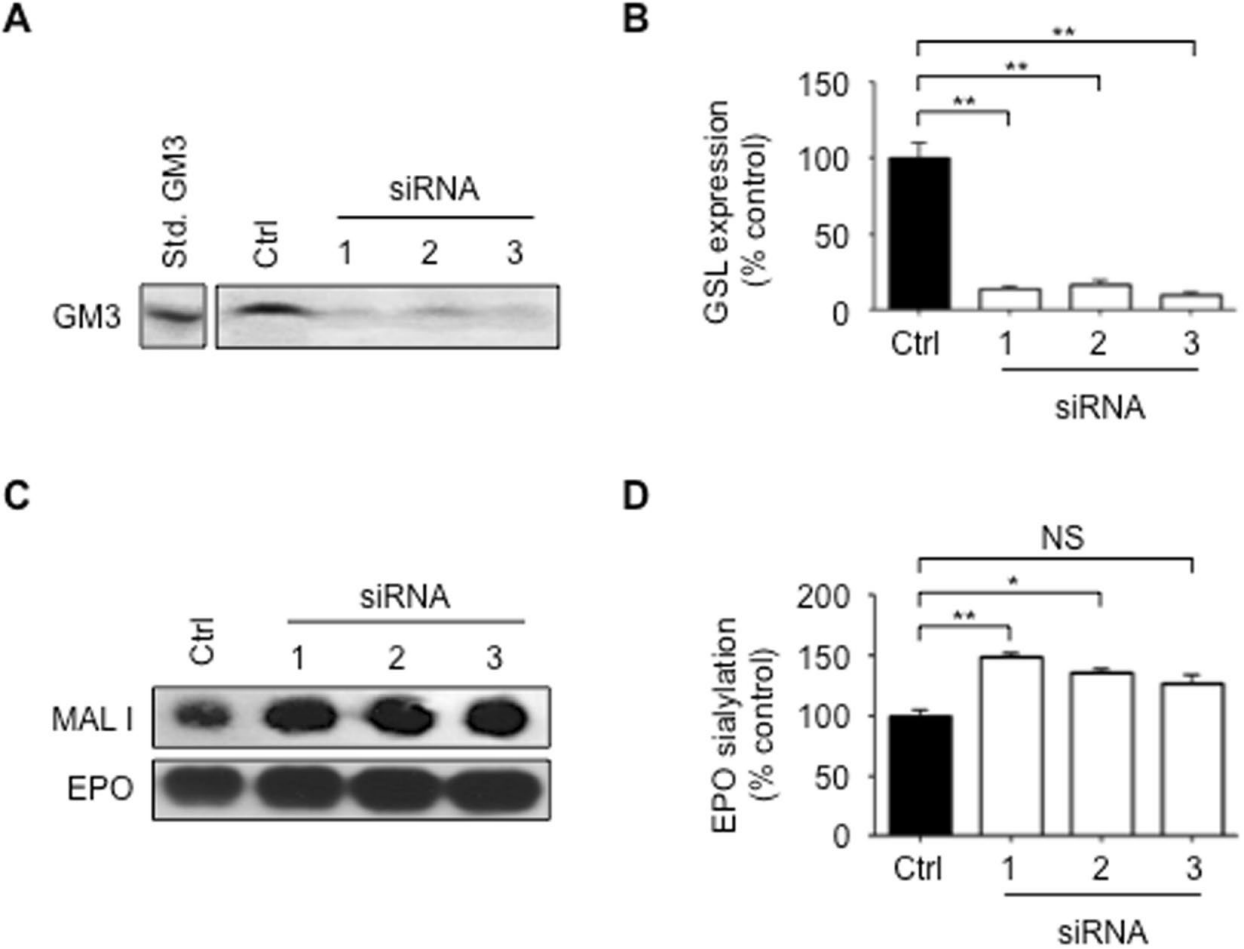
Transient repression of *UGCG* increases rhEPO sialylation. (**A**) Total GSLs extracted from cells were subjected to TLC followed by staining with orcinol/sulfuric acid. Lane 1, GM3 standard; Lane 2, GSLs extracted from EC2-1H9 cells; Lanes 3–5, GSLs extracted from EC2-1H9 cells treated with siRNAs #1–3; n = 3. (**B**) Quantification of total GSLs. n = 3. (**C**) rhEPO was purified using immunoprecipitation with an anti-EPO antibody. Sialylation was determined using MAL I lectin. (**D**) Quantification of data in (**C**); n = 3. *P < 0.05, **P < 0.01 (Two-tailed Student’s *t*-test). Data are presented as means ± S.E.M.

From a commercial perspective, the treatment of cells with chemical inhibitors is generally considered impractical because of the cost and potential for side effects. Instead, we hoped to develop a stable cell line that produces highly sialylated rhEPO as a consequence of its reduced expression of *UGCG*. As a first step in this direction, we designed three different small interfering RNAs to specifically target *UGCG* (Table SI). We were able to confirm that all three siRNAs significantly repress the expression of *UGCG* by EC2-1H9 CHO cells (Fig. S2A). Consistent with what we observed with EtDO-P4 treatment, all three siRNAs reduce the levels of ganglioside GM3 (Fig. 3A) and other GSLs (Fig. 3B). We next used an anti-EPO antibody to pull-down rhEPO from supernatant collected from these UGCG-depleted EC2-1H9 CHO cells. As expected, we found rhEPO purified from these UGCG-depleted CHO cells shows 50% more sialylation than rhEPO obtained from control cells (Fig. 3C and D).

### Stable UGCG depletion also enhances rhEPO sialylation

**Figure 4:**
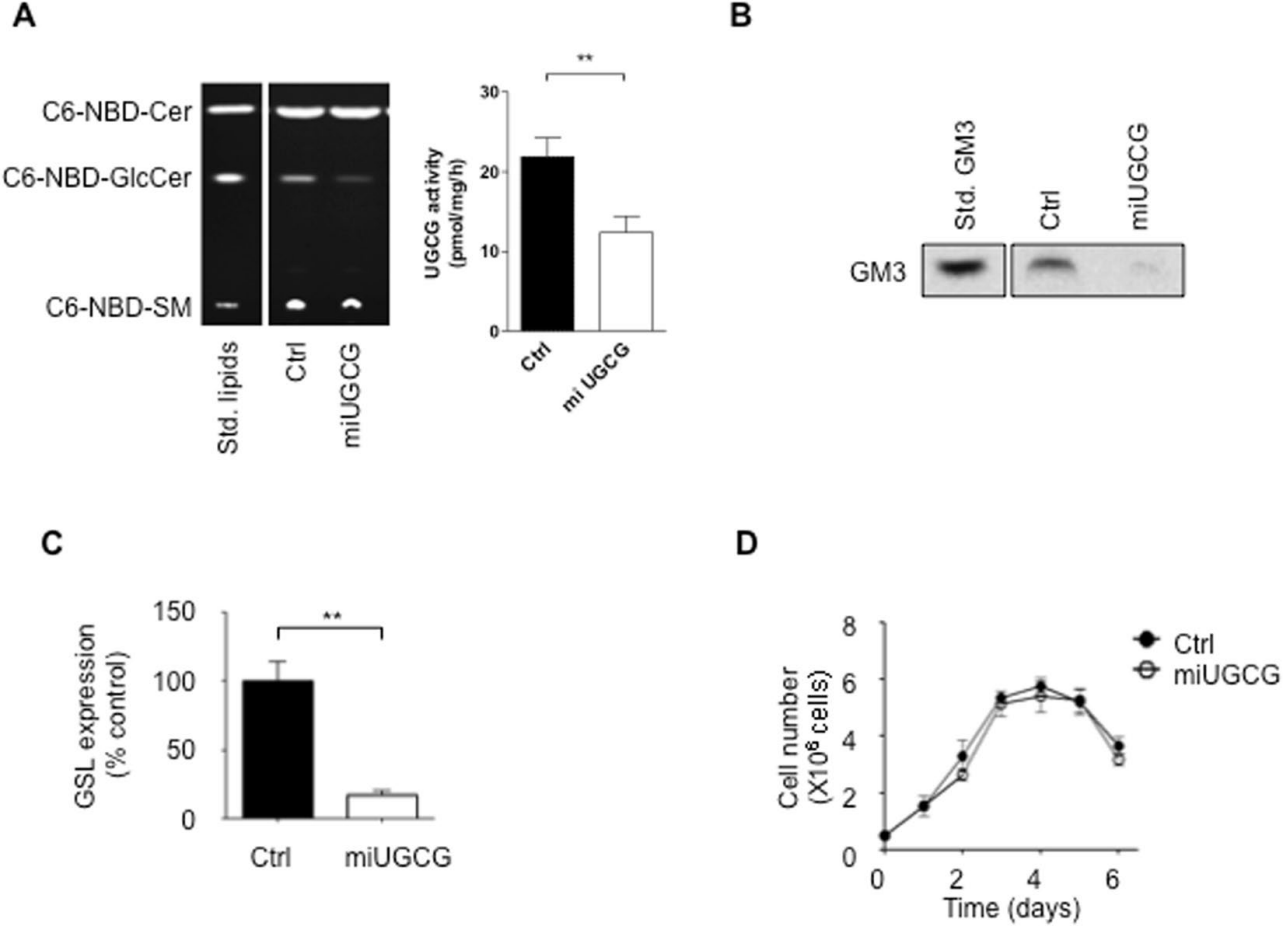
microRNA-mediated repression of *UGCG* in CHO cells. (**A**) Enzymatic activity of UGCG was determined by conversion of NBD-Cer to NBD-GlcCer on TLC. Left, Lane 1, Standard lipids; Lane 2, lipids extracted from control cells; Lane 3, lipids extracted from UGCG depleted cells. Right, Quantification of lipids was shown. (**B**) Total GSLs extracted from cells were subjected to TLC followed by staining with orcinol/sulfuric acid. Lane 1, GM3 standard; Lane 2, GSLs extracted from EC2-1H9 cells; Lane 3, GSLs extracted from EC2-1H9-miUGCG cells. n = 3. (**C**) Quantification of total GSLs. n = 3, **P < 0.01 (Two-tailed Student’s *t*-test). (**D**) Cell proliferation was determined for 6 days of culture. Closed circles, EC2-1H9 cells; Open circles, EC2-1H9-miUGCG. n = 3. Data are presented as means ± S.E.M.

Since transient knock-down of *UGCG* enhances rhEPO sialylation, we next attempted to establish a cell line that produces rhEPO in the context of stable *UGCG* depletion. All three siRNAs produce similar levels of rhEPO sialylation (see Fig. 3), so we designed an artificial miRNA expression vector, pcUGCG-miR-1, to target the same sequence as siRNA1. We transfected this vector into EC2-1H9 CHO cells and selected for a stable cell line. We confirmed that expression of the artificial miRNA reduces expression of *UGCG* as compared to control EC2-1H9 CHO cells (Fig. S2B). We also found that enzymatic activity of UGCG was inhibited in UGCG depleted cells as examined by the conversion of NBD-ceramide to NBD-glucosylceramide (Fig. 4A). The stable CHO cell line, which we named EC2-1H9-miUGCG, shows significantly reduced levels of ganglioside GM3 as well as other GSLs (Fig. 4B and C). Importantly, we confirmed that the rate of proliferation of EC2-1H9-miUGCG cells is similar to that of EC2-1H9 cells (Fig. 4D).

**Figure 5:**
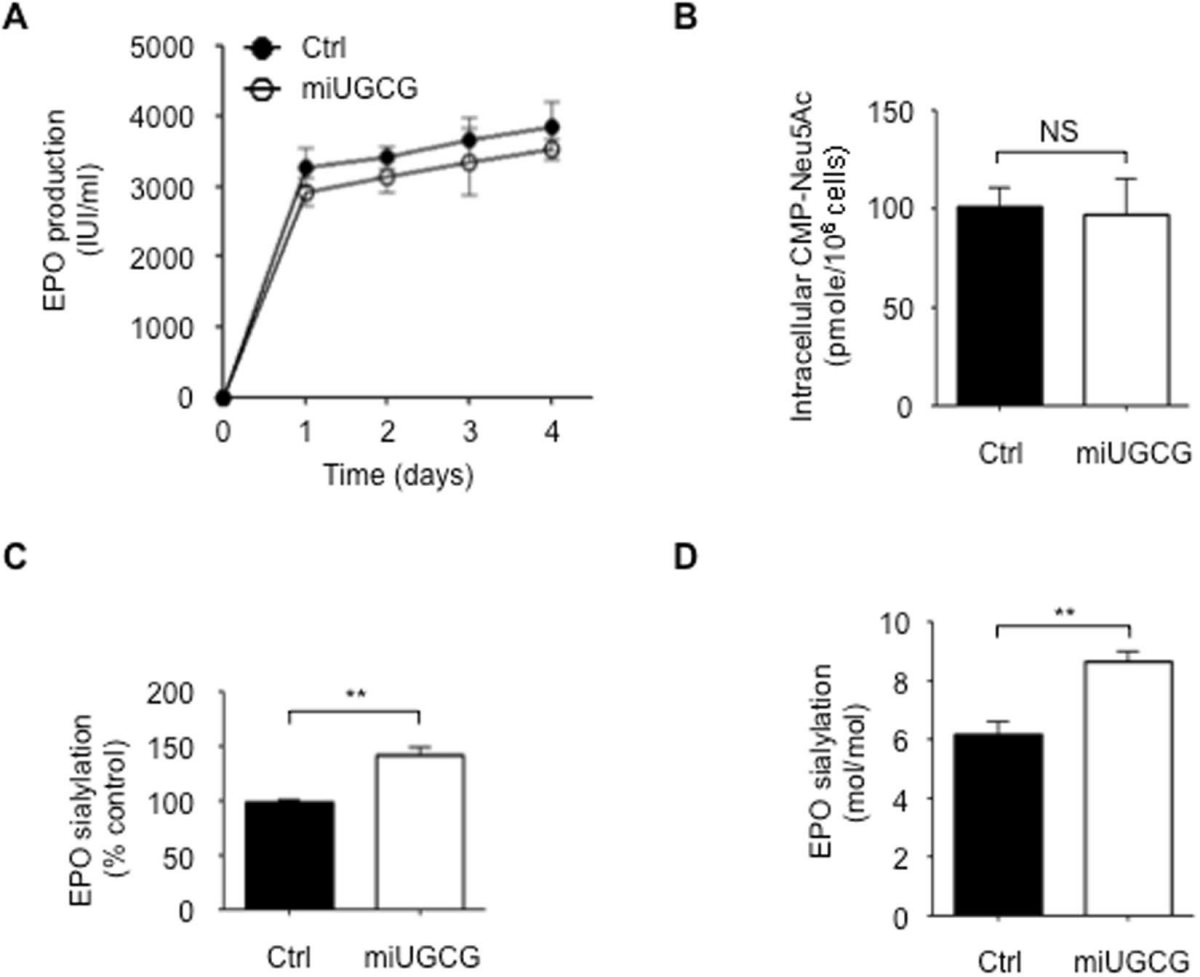
EC2-1H9-miUGCG cells produce rhEPO with increased levels of sialylation. (**A**) EPO production was determined for 4 days of culture. Closed circles, EC2-1H9 cells; Open circles, EC2-1H9-miUGCG cells. (**B**) Intracellular CMP-Neu5Ac levels were determined by HPLC. (**C**) rhEPO was purified using an immunoaffinity column with an anti-EPO antibody. Sialylation was determined using MAL I lectin. (**D**) Molar ratio of sialic acid on rhEPO. n = 3. **P < 0.01 (Two-tailed Student’s *t*-test); NS, not significant. Data are presented as means ± S.E.M.

To determine whether stable *UGCG* depletion enhances rhEPO sialylation, we allowed the EC2-1H9-miUGCG cells to produce rhEPO for 4 days (see Methods). We then used an anti-EPO antibody to purify rhEPO from the resulting supernatant using immunoaffinity chromatography. Both the EC2-1H9-miUGCG cells and the control EC2-1H9 cells produce similar levels of rhEPO (Fig. 5A). Although both types of cells show similar levels of intracellular CMP-Neu5Ac, EC2-1H9-miUGCG cells produce rhEPO with significantly more sialylation than that produced by EC2-1H9 cells (Fig. 5B and C). The similar levels of intracellular CMP-Neu5Ac suggest that the concentration of this metabolite in the sialylation pathway is maintained in a state of dynamic equilibrium. Since rhEPO contains one O-glycosylation site (Ser 126) and three N-glycosylation sites (Asn 24, Asn 38, and Asn 83), a single mole of rhEPO can be decorated with up to 14 moles of sialic acid^25^. Previously, we measured rhEPO sialylation using an o-phenylenediamine (OPD)-labeling technique coupled with reversed-phase HPLC with a C18 column^15,26^. Using this same approach, we found 8.5 moles of sialic acid per mole of rhEPO produced by EC2-1H9-miUGCG cells and only 6 moles of sialic acid per mole of rhEPO produced by EC2-1H9 cells (Fig. 5C). This suggests that the use of EC2-1H9-miUGCG cells may represent a novel method for generating large amounts of highly sialylated rhEPO.

### Analysis of N-linked glycan sialylation on rhEPO

**Figure 6:**
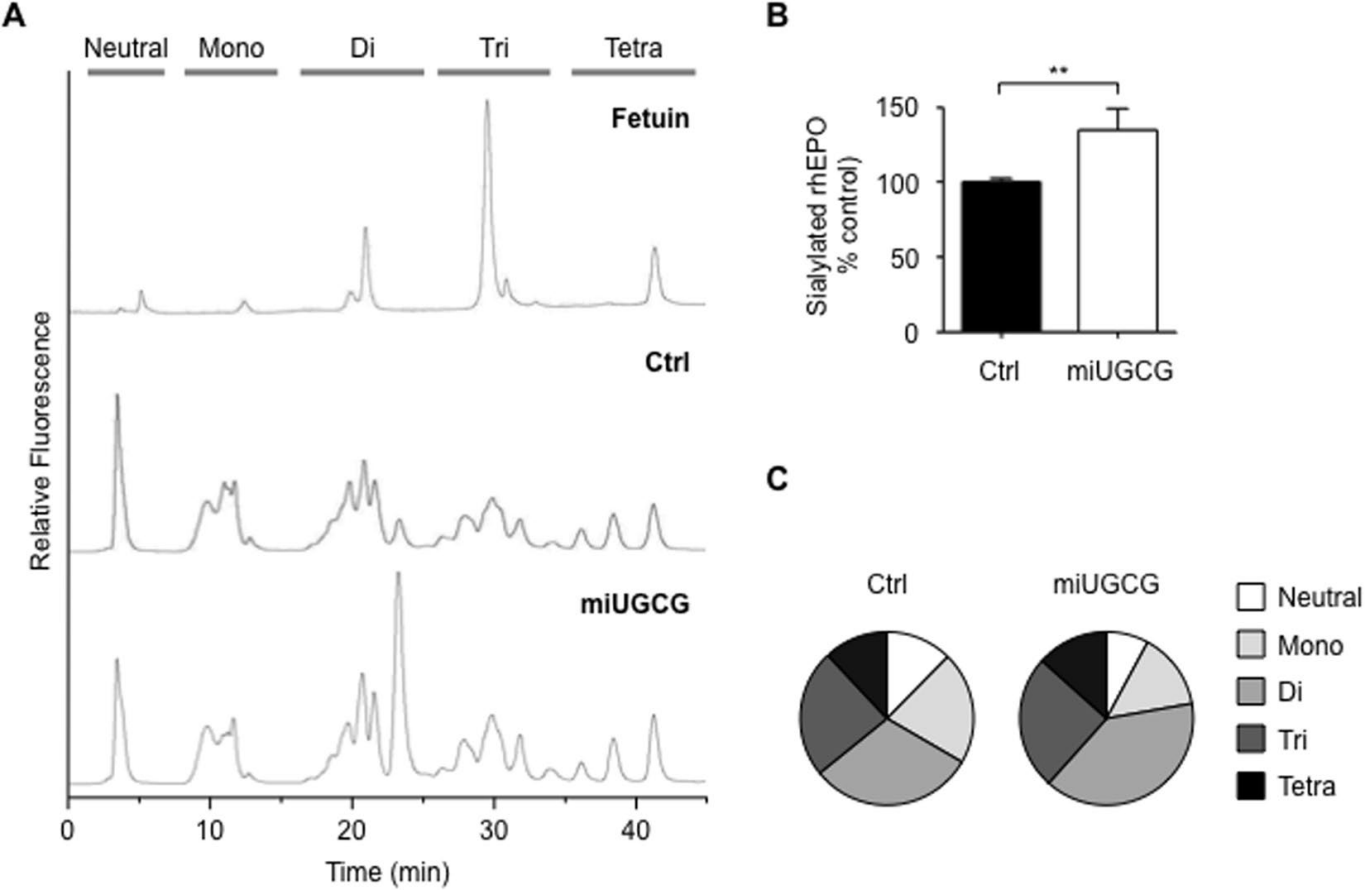
Analysis of sialylated N-linked glycans on rhEPO. (**A**) N-glycans released from rhEPO were labeled with 2-AB. 2-AB-labeled N-glycans were separated using a DEAE column by HPLC. 2-AB-labeled N-glycans of fetuin (top), rhEPO produced by EC2-1H9 cells (middle), and rhEPO produced by EC2-1H9-miUGCG cells (bottom). (**B**) Sialylated rhEPO determined in A. (**C**) Neutral, mono-, di-, tri-, and tetra-sialylated N-glycans of rhEPO quantified in A. n = 3. **P < 0.01 (Two-tailed Student’s *t*-test). Data are presented as means ± S.E.M.

To determine how stable depletion of UCGC affects the structure of the glycosylation chains on rhEPO, we used HPLC to analyze the N-linked glycans released from rhEPO after 2-aminobezaminde (2-AB) labeling^27^. We used an anion-exchange column (DEAE-5PW) to separate the 2AB-labeled N-linked glycans by their number of sialic acids and then estimated rhEPO sialylation by measuring the resulting peak area. For this sialylation profiling analysis, we used the 2-AB-labeled N-glycans of bovine fetuin as a sialylation standard (Fig. 6A). Consistent with the results described above (Fig. 5C and D), this technique confirmed that the rhEPO produced by EC2-1H9-miUGCG cells is more highly sialylated than that produced by EC2-1H9 cells (Fig. 6B). Specifically, we found EC2-1H9-miUGCG cells produce rhEPO with less neutral (6.9% vs. 12.4%) and mono-sialylated (14.4% vs. 21.0%) N-glycans compared to the control rhEPO produced by EC2-1H9 cells (Fig. 6C). Instead, the rhEPO produced by EC2-1H9-miUGCG cells shows significantly more di-sialylated N-glycans (40.8% vs. 30.7%) than the control rhEPO. The rhEPO produced by EC2-1H9-miUGCG cells shows similar levels of tri-sialylated (25.0% vs. 23.8%) and tetra-sialylated (12.9% vs. 12.1%) N-glycans compared to the rhEPO produced by EC2-1H9 cells (Fig. 6C).

## Discussion

Sialic acid is the terminal sugar on the glycosylation chains of most secreted glycoproteins, where it contributes to their function and stability^1,28,29^. In exploring the increased stability of recombinant therapeutic glycoproteins by regulating sialylation, we noted that it is also required for GSL biosynthesis^16^. We, therefore, hypothesized that inhibition of GSL biosynthesis may enhance the sialylation of rhEPO. First, we treated cells expressing rhEPO with a GSL biosynthesis inhibitor and confirmed the expected increase in rhEPO sialylation. Ultimately, this led us to establish the stable CHO cell line, EC2-1H9-miUGCG, which produces highly sialylated rhEPO owing to its reduced expression of *UGCG*. We recently discovered that we were able to increase intracellular CMP-Neu5Ac levels by co-expressing the CMP-Neu5Ac transporter CST with GNE (R263L/R266Q), bypassing any negative feedback inhibition^15^. Surprisingly, EC2-1H9-miUGCG cells show normal levels of CMP-Neu5Ac in the cytoplasm and in the Golgi (Fig. S3). This suggests that the increased intracellular CMP-Neu5Ac caused by inhibiting GSL biosynthesis is rapidly consumed in the sialylation of rhEPO, inducing a dynamic equilibrium.

Although we do not yet fully understand the intricacies of intracellular CMP-Neu5Ac dynamics, we used various approaches to confirm that EC2-1H9-miUGCG cells produce highly sialylated rhEPO. We found that the rhEPO produced by EC2-1H9-miUGCG cells has 10.2% more di-sialylated N-glycans than that produced by EC2-1H9 cells, but less neutral and mono-sialylated N-glycans. Although we were previously able to attribute the enhanced sialylation of the rhEPO produced by EC2-1H9-CTSTrEKm cells to tetra-sialylated N-glycans^15^, the rhEPO produced by EC2-1H9-miUGCG cells does not show any changes in the levels of tri- and tetra-sialylated N-glycans. This means it may be possible to improve the sialylation of rhEPO produced by EC2-1H9-CTSTrEKm cells even further via *UGCG* depletion. In addition, because the rate at which proteins transit the Golgi complex affects glycosylation^30^, it may also be possible to enhance rhEPO sialylation by slowing its passage through the Golgi.

In this study, we have determined that glycoprotein sialylation can be enhanced via the inhibition of GSL biosynthesis. We extended this finding by establishing a stable CHO cell line that produces highly sialylated rhEPO by virtue of its reduced expression of the GSL biosynthesis enzyme UGCG. In summary, we have developed a novel approach that improves the efficacy of rhEPO, a therapeutically important recombinant glycoprotein, by modulating the metabolic flux of intracellular CMP-Neu5Ac.

## Methods

### Inhibition of GSL biosynthesis by EtDO-P4

EtDO-P4 (D-threo-1-(3′,4′-ethylenedioxy)-phenyl-2-palmitoylamino-3-pyrrolidino-1-propanol), a nanomolar inhibitor of GSL synthesis, was provided by Dr. James A. Shayman (Department of Internal Medicine, University of Michigan, Michigan, USA). For the EtDO-P4 treatment experiments, 5 × 10^6^ cells were cultured on 100 mm plates in MEM-α (Minimum Essential Medium Eagle-α; Gibco, Grand Island NY) supplemented with 10% dFBS (dialyzed fetal bovine serum; Gibco) for 24 hours^31^. Cells were washed twice with serum-free medium (CHO-S-SFM II; Gibco) before culturing them for an additional 48 hours in serum-free medium containing 1 µM EtDO-P4.

### Western blotting

rhEPO containing culture media was subjected to SDS-PAGE, followed by transferring to a PVDF membrane (Millipore Corp., Bedford, MA). After blocking with 5% BSA in TBS-T (140 mM NaCl, 10 mM Tris-HCl, with 0.05% Tween 20, pH 8.0) for 1 hour at room temperature, the PVDF membrane was incubated with biotinylated lectins (Wheat Germ Agglutinin; WGA, Maackia Amurensis leukoagglutinin I; MAL I) at 4 °C for 16 hours. After rinsing three times for 5 min using TBS-T, the membrane was incubated with ExtrAvidin-peroxidase (Sigma) at room temperature for 1 hour, followed by developing using ECL kit (Thermo Scientific; Rockford, IL). For reblotting, the membrane was stripped at room temperature for 1 hour with stripping buffer (Candor Bioscience GmbH, Weissensberg, Germany). PVDF membrane was rinsed 5 times with T-TBS. The membrane was blocked using 5% BSA in TBS-T, and then incubated with 1:1000 diluted anti-EPO antibody (Santa Cruz, CA). After washing three times with TBS-T for 5 min, the membrane was incubated with 1:5000 diluted HRP-labeled anti-mouse IgG antibody (Santa Cruz) at room temperature for 1 hour. The membrane was developed using ECL solution (Thermo Scientific; Rockford, IL).

### Depletion of UGCG expression using siRNA and miRNA

Three siRNA sequences (siRNA1, siRNA2 and siRNA3) which targets Chinese hamster *UGCG* mRNAs (GenBank accession no. NM_001246692) were obtained from Invitrogen (Carlsbad, CA). The oligonucleotides are listed in Table SI. 10 μM of each *UGCG*-specific siRNA was transfected to the EC2-1H9 cells using Lipofectamine™ RNAiMAX reagent (Invitrogen, Carlsbad, CA) according to the manufacturer’s instruction. To establish stable cell line which prevents the expression of UGCG, miRNA expression vector was prepared according to the sequence of siRNA1 (see Table S1). In brief, paired oligonucleotides were annealed in buffer (30 mM HEPES-KOH, pH 7.4, 150 mM KCl, 2 mM MgCl2) at 90 °C for 1 min followed by cooling down. To prepare the pcUGCG-miR-1 vector, DNA fragment was inserted into the pcDNA^TM^ 6.2-GW/EmGFP-miR vector (Invitrogen, Carlsbad, CA), which contains a blasticidin gene for the selection.

Total RNAs extracted from CHO cells with TRIzol^®^ (Invitrogen, Carlsbad, CA) were used to prepare cDNA by which extracted RNAs were reversed transcribed into using AccuPower RT-PCR PreMix (Bioneer) according to the manufacturer’s instructions. Expression level of UGCG was examined by RT-PCR using following primers shown 5′ to 3′: forward (CTC GAG ATG GCG CTG CT); reverse (TCT AGA TTA TAC ATC TAG GAT TTC CTC TGC). The amplified cDNAs were separated on 0.8% agarose gel which was visualized by ethidium bromide (EtBr) staining.

### Establishment of UGCG depleted CHO cell line

EC2-1H9 cells, which are CHO cells that produce recombinant human EPO, were provided by Dr. Hyo Jeong Hong (Antibody Engineering Research Unit, Korea Research Institute of Bioscience and Biotechnology, Yuseong-gu, Daejeon, Korea). Cells were maintained in MEM-α (Gibco) supplemented with 10% (v/v) dFBS (Gibco), 3.5 g/L glucose, 20 nM MTX (methotrexate; Sigma), and 1% (v/v) Ab-Am (antibiotic-antimycotic solution; Gibco) in a humidified atmosphere containing 5% CO_2_ at 37 °C. The pcUGCG-miR-1 was transfected into EC2-1H9 cells using Lipofectamine™ 2000 (Invitrogen, Carlsbad, CA). Transfected cells were selected by blasticidin (Sigma) for 2 weeks.

### GSL extraction, thin-layer chromatography (TLC), and immunostaining

GSL extraction, TLC analysis, and immunostaining experiments were performed as previously described^32^. Briefly, GSLs were extracted from confluent CHO cells using chloroform/methanol (C/M; 2:1, v/v) and isopropanol/hexane/water (IPA/H/W; 55:25:20, v/v/v) under sonication for 30 min. GSL extracts were dissolved in 0.1 M NaOH/MeOH and incubated at 40 °C for 2 hours, followed by neutralization using 1 N HCl. Hexane was added and incubated at room temperature for 5 min. The lower layer was evaporated under N2 stream and resuspended using distilled water. The solution was subjected to SepPak C_18_ cartridge (Varian, Palo Alto, CA). After rinsing the column using distilled water, total GSLs were eluted using C/M. Total GSLs were determined on TLC (Silica Gel 60 F-254, Merck, Whitehouse Station, NJ) in C/M/0.2% CaCl_2_ (55:40:10, v/v/v), followed by staining using 2% orcinol in 2 M H_2_SO_4_.

### Determination of the enzymatic activity of UGCG

Enzymatic activity of UGCG was determined as previously established^33,34^. In brief, cells (4 × 10^7^) in TBS containing protease inhibitor (Roche, Basel, Switzerland) were lysed using sonication. Cell lysate (200 µg) was incubated in reaction buffer [50 mM Tris-HCl pH 7.5, 1 mM EDTA, 50 pmol of C6-NBD-Cer (ThermoFisher Scientific, Waltham, MA), 500 µM of UDP-Glc, 6.5 nmol of lecithin (Wako, Osaka, Japan)] at 37 °C for 60 min. Lipids were extracted by Folch method using 200 µl of chloroform/methanol (2:1, v/v) and 5 µl of 500 µM KCl. After centrifugation, the organic phase was dried using centrifugal concentrator. Samples were suspended using 15 µl of chloroform/methanol (2:1, v/v) and subjected to the TLC (Silica Gel 60, Merck) which was developed with chloroform/methanol/water (65:25:4, v/v/v). Lipids were determined by LED transilluminator at 470 nm. C6-NBD-Cer, C6-NBD-GlcCer, C6-NBD-SM were quantified using a Shimadzu CS-9300 chromatoscanner (excitation: 475 nm, emission: 525 nm).

### Isolation of the Golgi complex

Golgi membrane was extracted from CHO cells as previously described^35^. Briefly, 1.0 × 10^8^ CHO cells were resuspended with homogenizing buffer (0.25 M sucrose, 10 mM Tris-HCl, pH 7.4) and homogenized using ball-bearing homogenizer with 22 µm ball. The solution was subjected to sucrose gradient centrifugation at 110,000 x g for 2.5 hours. Golgi membrane was collected at the 29%/35% sucrose interface using a syringe with 18G gauge needle, and determined the protein concentration using the Quant-iT™ protein assay kit (Invitrogen).

### Determination of intracellular CMP-sialic acid levels in CHO cells

The concentration of intracellular CMP-Neu5Ac in CHO cells was examined as previously described^36^. Briefly, 1.0 × 10^6^ cells were lysed in cold 75% (v/v) ethanol using a cell disruptor. The soluble fraction was separated using centrifugation at 13,000 rpm at 4 °C for 10 min. The lyophilized CMP-Neu5Ac was dissolved with 120 μl of 40 mM phosphate buffer (pH 9.2), followed by centrifugation. The supernatant was filtered using a centricon (MWCO, 10,000) and subjected to CarboPac PA-1 column (Dionex, Sunnyvale, CA). CMP-sialic acid was determined by Abs_260_ absorbance detector (model 486; Waters). The concentration of intracellular CMP-Neu5Ac was normalized to cell number.

### Production of rhEPO and determination of its sialic acid level

5.0 × 10^6^ of EC2-1H9 cells or the engineered cells were seeded in T175 culture flask containing MEM-α supplemented with 10% (v/v) dFBS, 3.5 g/L glucose, 1% (v/v) Ab-Am solution, and 20 nM MTX. The culture medium was replaced with serum-free medium (CHO-S-SFM II; Gibco) in 3 days. After 2 days, the culture medium was collected, filtered using 0.45 μm filter (Sartorius, Göttingen, Germany), and dialyzed against phosphate buffer saline (PBS, pH 7.4) at 4 °C, overnight. To purify rhEPO, cultured medium was subjected to EPO Purification Gel (MAIIA Diagnostics, Uppsala, Sweden) according to the manufacturer’s instructions. Purified rhEPO was dialyzed against distilled water at 4 °C, overnight. The concentration was measured using a Quant-iT™ protein assay kit (Invitrogen) and stored at −80 °C until use.

The sialic acid level of the purified rhEPO was examined using the OPD-labeling method as previously described^26^. Briefly, sialic acid moieties from the purified rhEPO were released using 0.5 M NaHSO_4_ at 80 °C for 20 min. Released sialic acid was labeled with OPD (*o*-phenylenediamine-2HCl; Sigma) at 80 °C for 40 min. The level of OPD-labeled sialic acid was determined using C18 reversed-phase column (Shim-pack CLC-ODS; Shimadzu, Kyoto, Japan) with 474 scanning fluorescence detector (excitation at 230 nm and emission at 420 nm, Waters).

### Profiling the sialylation of rhEPO’s N-linked glycans

The purified rhEPO (50 μg) was denatured by heating at 95 °C for 5 min. N-glycans were released from rhEPO by incubation with PNGase F (Promega) at 37 °C for 3 hours and purified using GlycoClean R cartridges (Glyko; ProZyme, Hayward, CA). Purified N-glycans were labeled with 2-AB using 2-AB labeling kit (Glyko; ProZyme, Hayward, CA). The sample was subjected to an anion-exchange column (TSKgel DEAE-5PW, 7.5 mm × 75 mm; Tosoh, Tokyo, Japan) as described previously (Llop *et al*. 2007). These included solvent A (50% 0.5 M ammonium formate (pH 4.5)/acetonitrile (ACN)/distilled water (5:2:3, v/v/v)) and solvent B (20% (v/v) ACN in distilled water). 2AB-labeled N- glycans were separated in a linear gradient of buffer B from 0% (v/v) to 100% (v/v) over 45 min at a flow rate of 0.4 mL/min at 30 °C. 2-AB N-glycans of bovine fetuin (Glyko) were used as a standard.

## Electronic supplementary material

Dataset 1
