# Supplementary material for: Enhancing the sialylation of recombinant EPO produced in CHO cells via the inhibition of glycosphingolipid biosynthesis
Source: Sci Rep. 2017 Oct 12;7:13059. doi: 10.1038/s41598-017-13609-4 (PMC5638827; doi:10.1038/s41598-017-13609-4)
Supplement: Supplementary file 1 — Dataset 1 [file 41598_2017_13609_MOESM1_ESM.pdf]

Supplementary information

Enhancing the sialylation of recombinant EPO produced in CHO cells via the inhibition of glycosphingolipid biosynthesis

Chan-Yeong Kwak[1,4], Seung-Yeol Park[2,4,5], Chung-Geun Lee[1], Nozomu Okino[3], Makoto Ito[3], and Jung Hoe Kim[1,5]

[1]*Department of Biological Sciences, Korea Advanced Institute of Science and Technology, 335 Gwahangno, Yuseong-gu, Daejeon 305-701, Republic of Korea.*

[2]*Division of Rheumatology, Immunology and Allergy, Brigham and Women's Hospital, and Department of Medicine, Harvard Medical School, Boston, MA 02115 USA.*

[3]*Department of Bioscience and Biotechnology, Graduate School of Bioresource and Bioenvironmental Sciences, Kyushu University, 6-10-1, Hakozaki, Higashi-ku, Fukuoka 812-8581, Japan.*

[4]Equal contributions

[5]Address correspondence to:

Jung Hoe Kim

Tel: +82-42-350-2614 Email: kimjh@kaist.ac.kr

Seung-Yeol Park

Tel: +1-617-525-1073 Email: spark@bwh.harvard.edu

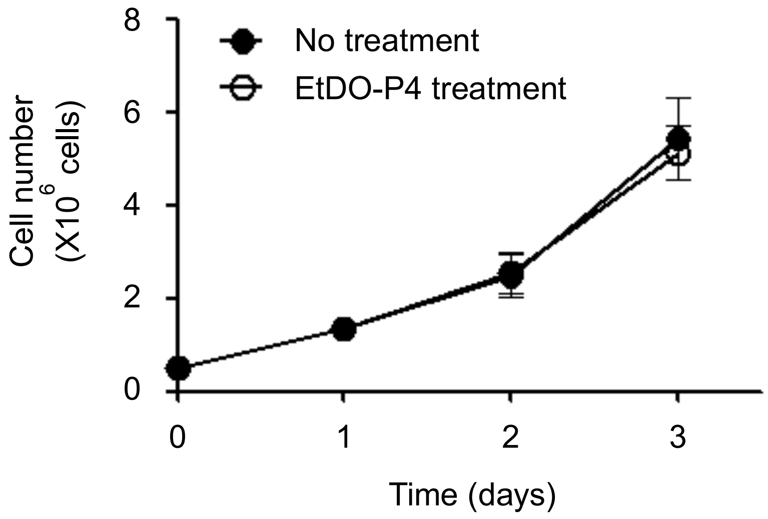

Figure S1. EtDO-P4 does not affect cell growth. Cell proliferation measured during EtDO-P4 treatment. Closed circles, EC2-1H9 cells; Open circles, EC2-1H9 cells treated with EtDO-P4. n = 3. Data are presented as means ± S.E.M..

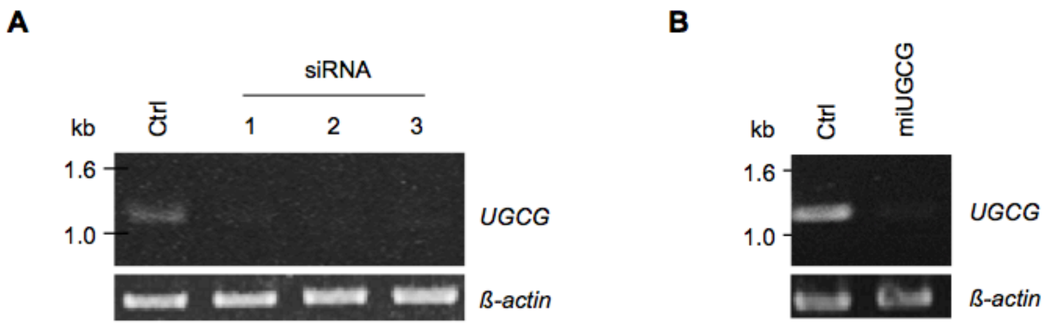

Figure S2. *UGCG* knock-down. A, siRNA-mediated knock-down of *UGCG*. Lane 1, EC2-1H9 cells; Lanes 2–4, EC2-1H9 cells depleted of *UGCG* by siRNAs #1–3. B, miRNA-mediated repression of *UGCG*. Lane 1, EC2-1H9 cells; Lane 2, EC2-1H9-miUGCG cells; *β-actin* was used as the loading control.

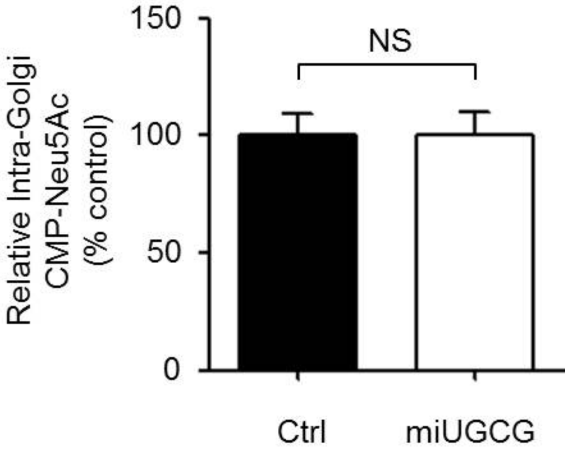

Figure S3. Quantification of intra-Golgi CMP-Neu5Ac. CMP-Neu5Ac extracted from isolated Golgi was separated on a CarboPac PA1 column; n = 2; NS, not significant. Data are presented as means ± S.E.M..

Table SI. Oligonucleotides used in this study.

Name	Oligonucleotide	Oligonucleotide sequence	Position in cDNA
siRNA1	Sense	5'-CAUUAUGGGACCCUACUAUAA-3'	1100
	Anti-sense	5'-UUAUAGUAGGGUCCCAUAAUG-3'	
siRNA2	Sense	5'-GUGUUCAGAUGGGAUAUCAUG-3'	928
	Anti-sense	5'-CAUGAUAUCCCAUCUGAACAC-3'	
siRNA3	Sense	5'-UGAUAGCCUUUGCUCAGUACA-3'	677
	Anti-sense	5'-UGUACUGAGCAAAGGCUAUCA-3'	
